# Plausible Biological Interactions of Low- and Non-Calorie Sweeteners with the Intestinal Microbiota: An Update of Recent Studies

**DOI:** 10.3390/nu12041153

**Published:** 2020-04-21

**Authors:** Julio Plaza-Diaz, Belén Pastor-Villaescusa, Ascensión Rueda-Robles, Francisco Abadia-Molina, Francisco Javier Ruiz-Ojeda

**Affiliations:** 1 Department of Biochemistry and Molecular Biology II, School of Pharmacy, University of Granada, 18071 Granada, Spain; 2 Institute of Nutrition and Food Technology “José Mataix”, Center of Biomedical Research, University of Granada, Avda. del Conocimiento s/n., 18016 Armilla, Granada, Spain; 3 Instituto de Investigación Biosanitaria IBS.GRANADA, Complejo Hospitalario Universitario de Granada, 18014 Granada, Spain; 4 LMU–Ludwig-Maximilians-University of Munich, Division of Metabolic and Nutritional Medicine, von Hauner Children’s Hospital, University of Munich Medical Center, 80337 Munich, Germany; 5 Institute of Epidemiology, Helmholtz Zentrum München–German Research Centre for Environmental Health, 85764 Neuherberg, Germany; 6 Department of Cell Biology, School of Sciences, University of Granada, 18071 Granada, Spain; 7 RG Adipocytes and metabolism, Institute for Diabetes and Obesity, Helmholtz Diabetes Center at Helmholtz Center Munich, 85764 Neuherberg, Munich, Germany

**Keywords:** nonnutritive sweeteners, sweetening agents, gut microbiota

## Abstract

Sweeteners that are a hundred thousand times sweeter than sucrose are being consumed as sugar substitutes. The effects of sweeteners on gut microbiota composition have not been completely elucidated yet, and numerous gaps related to the effects of nonnutritive sweeteners (NNS) on health still remain. The NNS aspartame and acesulfame-K do not interact with the colonic microbiota, and, as a result, potentially expected shifts in the gut microbiota are relatively limited, although acesulfame-K intake increases Firmicutes and depletes *Akkermansia muciniphila* populations. On the other hand, saccharin and sucralose provoke changes in the gut microbiota populations, while no health effects, either positive or negative, have been described; hence, further studies are needed to clarify these observations. Steviol glycosides might directly interact with the intestinal microbiota and need bacteria for their metabolization, thus they could potentially alter the bacterial population. Finally, the effects of polyols, which are sugar alcohols that can reach the colonic microbiota, are not completely understood; polyols have some prebiotics properties, with laxative effects, especially in patients with inflammatory bowel syndrome. In this review, we aimed to update the current evidence about sweeteners’ effects on and their plausible biological interactions with the gut microbiota.

## 1. Introduction

Excessive sugar consumption has become an important public health concern due to its adverse effects on health and metabolic consequences such as obesity, insulin resistance, metabolic syndrome, cardiovascular diseases, and type 2 diabetes. One century ago, sweetening agents or sweeteners—sugar substitutes that mimic the sweet taste—emerged as an alternative to sucrose and glucose–fructose syrups consumption to reduce energy intake [1,2]. However, the impact of sugar consumption on health continues to be a controversial topic in relation to its effects on metabolic disease [3]. Some contradictory results were published in 2015 about sweeteners and gut microbiota. Suez et al. concluded that some sweeteners may affect the human microbiome, and consequently more studies are needed [4]. In contrast, Frankelfeld et al. [5] reported no differences in median bacterial abundance across consumers and non-consumers of sweeteners.

Sweeteners are between several hundred to thousands of times sweeter than sucrose and they do not contain too many calories. They include nonnutritive sweeteners (NNS), which have a higher sweetening intensity than other sweeteners, such as acesulfame K (ace-K), advantame, aspartame, aspartame–acesulfame salt, cyclamate, neohesperidin dihydrochalcone, neotame, saccharin, steviol glycosides (including 10 different glycosides), sucralose, and thaumatin, low-calorie sweeteners (LCS), such as polyols or sugar alcohols and other new sugars which are low-digestible carbohydrates derived from the hydrogenation of their sugar or syrup sources. Polyols are around 25%–100% as sweet as sugar and include erythritol, hydrogenated starch hydrolysates (sometimes listed as maltitol syrup, hydrogenated glucose syrup, polyglycitol, polyglucitol, or simply HSH), isomalt, lactitol, maltitol, mannitol, sorbitol, and xylitol. All of them are considered safe for human consumption as long as they are consumed within the acceptable daily intake [6]. This safety was claimed by the European Food Safety Authority (EFSA) except for cyclamate, which is not approved by the US Food and Drug Administration [1,7,8].

In 2019, we reviewed the effects of sweeteners on the gut microbiota, considering both experimental studies and clinical trials, and we reported that, among NNS, only saccharin and sucralose shift the populations of the gut microbiota, although more human studies are needed to clarify those observations. Within nutritive sweeteners (NS), only stevia extracts seem to affect gut microbiota composition, while some polyols, such as isomalt and maltitol which can reach the colon, increase *Bifidobacterium* in healthy subjects and might act as prebiotics. Besides, lactitol can decrease *Bacteroides*, *Clostridium*, coliforms, and *Eubacterium*, increasing butyrate and IgA secretion in humans [1]. Thus, we concluded that still more studies are needed; however, as the plausible biological interaction between sweeteners and intestinal microbiota has not been reported elsewhere, we aimed to review and update the current knowledge about sweeteners and gut microbiota interactions in humans.

A comprehensive literature search was conducted in PubMed, Embase^®^, and Scopus using different combinations of the following keywords: aspartame, acesulfame-K, cyclamate, sucralose, saccharin, steviol glycosides, erythritol, isomalt, lactitol, maltitol, sorbitol, mannitol, xylitol, and microbiota, with special attention and interest to what was published from February 2018 to March 2020.

## 2. Biological Plausibility: Which Low- and Non-Calorie Sweetener (LNCS) Could Potentially Affect the Colonic Microbiota?

Biological plausibility is one component of a method of reasoning that can establish a cause-and-effect relationship between a biological factor and a particular disease or adverse effect based on assessing the strength of evidence, since the work of Bradford Hill [9]. Here, we will assess biological plausibility between different sweeteners and gut microbiota composition. Although we usually refer to the different low- and non-calorie sweeteners (LNCS) as if they were a single molecule, it is well known that they do not share their absorption, distribution, metabolism, and excretion (ADME) profiles. Therefore, the extrapolation of the effect of a particular LNCS on the intestinal microbiota to all LNCS is unappropriated. These differences are crucial to understanding if each LNCS has the potential to alter the intestinal microbiota, directly or indirectly. For more detailed information on the metabolic fate of each LNCS beyond its relationship with the colonic microbiota, the excellent review by Magnuson et al. (2016) [10] can be consulted.

### 2.1. Effects of Non-Nutritive Low-Calorie Sweeteners on the Gut Microbiota

#### 2.1.1. Aspartame

Aspartame is a methyl ester of a dipeptide composed of L-phenylalanine and aspartic acid. When ingested, this dipeptide undergoes enzymatic hydrolysis in the gastrointestinal lumen and in the cells of the internal intestinal mucosa (by peptidases and intestinal esterases), so that virtually no aspartame enters the general circulation [11,12]. Hence, aspartame as an intact molecule cannot interact directly with the colonic microbiota. The three digestion products (aspartic acid, L-phenylalanine, and methanol) are rapidly absorbed in the duodenum and jejunum [12], reaching the systemic circulation without passing through the colon [10]. These degradation products are presented in the same way as when they are absorbed from vegetables, fruits, dairy, or meat, and at much lower concentrations than when they are derived from such foods [11]. These products follow their usual metabolic pathways.

Methanol enters the portal circulation into the liver and, by the enzymatic action of alcohol dehydrogenase, is metabolized to formaldehyde, which in turn, by the action of formaldehyde dehydrogenase, is oxidized to formic acid. Formic acid can be eliminated by the respiratory tract as carbon dioxide or excreted into the urine [10,11]. Aspartate undergoes a transamination reaction in the enterocytes, becoming oxalacetate. Oxalacetate and aspartate are interconverted in the body and can participate in the urea cycle and gluconeogenesis in the liver. Excess aspartate is eliminated in the urine [10]. Phenylalanine is absorbed in the gastrointestinal tract mucosa. It enters the liver through portal circulation, where, by the action of phenylalanine hydroxylase, can be converted into tyrosine. Phenylalanine that reaches the systemic circulation can be distributed throughout the body [11]. Its excess is excreted in the urine [13].

Based on the abovementioned information, the finding of a positive association between intake of aspartame and alteration of the colonic microbiota of rodents could be in fact due to the effect of what the animals stop eating rather than to the effect of aspartame intake itself. This last concept applies to all LNCS. However, a recent study carried out in female Sprague Dawley rats subjected to a high-fat/sucrose (HFSD), a HFSD + aspartame (5–7 mg kg^−1^ day^−1^), or a HFSD + stevia (2–3 mg kg^−1^ day^−1^) diet showed an increase of body fat in the offspring at weaning following maternal consumption of aspartame and stevia in the HSFD. In addition, glucose tolerance was altered, particularly with aspartame. *Akkermansia muciniphila* and *Enterobacteriaceae* concentrations were higher in mothers compared with their offspring. Regarding the cecal microbiota, a reduced abundance of *Enterococcaceae*, *Enterococcus*, and *Parasutterella* and an increased abundance of *Clostridium* cluster IV were found in the aspartame group. Moreover, fecal transplantation from offspring to germ-free mice produced an altered gut microbiota, causing impaired adiposity and glucose tolerance. In addition, increased concentrations of *Porphyromonadaceae* in males and females obese–aspartame and obese–stevia offspring were found [14]. In contrast, in the study by Suez et al., food intake in mice assigned to a water group with LNCS (aspartame, sucralose, and saccharin) was reduced by up to 50%. It is known that dietary factors are key determinants of the composition of the intestinal microbiota; indeed, differences in both total caloric intake and the type of food consumed can lead to a different microbial composition [15,16,17].

Thus, the intestinal microbiota might have been altered by a reduced consumption of fiber, protein, fat, and carbohydrates; therefore, it seems uncertain that the reported change in the intestinal microbiota was caused by the LNCS, and the changes that diet per se may provoke in the intestine should be considered. Nonetheless, there are studies that reveal possible modifications of the microbiota due to the use of aspartame. The study by Mahmud et al. analyzes the combined and individual effects of the administration of low concentrations of aspartame and Ace-K. Induction of *Escherichia coli* growth and expression of some important genes which may be related to its colonization in the gut were observed [18]. In another study with human fecal samples, aspartame administration significantly increased *Bifidobacterium* and *Blautia coccoides* growth and decreased the *Bacteroides*/*Prevotella* ratio; nevertheless, the aspartame-based sweetener used in this study was rich in maltodextrin, thus, the authors did not study the effect of aspartame alone [19].

#### 2.1.2. Potassium Acesulfame (Ace-K)

After its intake, Ace-K is absorbed almost completely in the small intestine as an intact molecule and distributed by the blood to different tissues. Without undergoing any metabolization, more than 99% of Ace-K is excreted in the urinary tract within the first 24 h, with less than 1% being eliminated in the feces [10,20]. The minimum amount of Ace-K ingested, its rapid absorption, and its urinary excretion causes the Ace-K concentration that reaches the fecal or colonic bacteria to be negligible [10,21]. Therefore, it is extremely unlikely that this LNCS could have a direct effect on the colonic microbiota [16]. However, some studies have reported small shifts in the gut microbiota composition following Ace-K intake.

A cross-sectional study was conducted in humans and showed no modifications in the intestinal microbiota nor significant differences by sex, contrary to the study conducted in rats by Bian X et al. [22]. Other studies also indicate that Ace-K causes changes in the microbiota and their metabolites, such as butyrate and pyruvate [22,23]. The study carried out by Uebanso et al. suggests that the daily intake of maximum adequate diary intake (ADI) levels of Ace-K does not affect the relative amount of the *Clostridium* cluster XIVa in the fecal microbiome [24]. In contrast, a study in mice that received 150 mg kg^−1^ of Ace-K by free drinking during 8 weeks, showed that lymphocyte recruitment was increased, with augmented expression of inflammatory cytokines and adhesion molecules [25]. Recent studies in rats indicate that administration of a mixture of sucralose and Ace-K at concentrations near the upper limit of ADI for human consumption during mice pregnancy has consequences on the progeny, causing metabolic and microbiome alterations. The authors observed an increase in *Firmicutes* and a depletion of *A. muciniphila*, which is a beneficial bacterium inversely correlated with fat mass gain, type 1 diabetes, and inflammatory bowel syndrome (IBS) [26]. The researchers also indicated an increase in the variety of species in the microbiota; however, *A. muciniphila* was significantly depleted, suggesting that the divergence between mothers’ and pups’ microbiomes was due to increasing NNS concentrations [27].

Regarding the bacteriostatic effect of Ace-K, this sweetener shows a strong inhibitory effect on the growth of *E. coli* HB101 and *E. coli* K-12 [28]. In contrast, using a concentration of Ace-K of 2.5 mg/mL, the result was an induction in *E. coli* growth, whereas the growth stimulation decreased gradually when higher concentrations of sweetener were used [18].

#### 2.1.3. Cyclamate

Cyclamate is the sodium or calcium salt of cyclamic acid (cyclohexanesulfamic acid), which itself is prepared by the sulfonation of cyclohexylamine and is eliminated in the feces [29]. In a study carried out by Vamanu et al. [30], the authors determined the effect of sweeteners on the microbiota pattern using an in vitro model. In this study, the total quantity of synthesized short-chain fatty acids (SCFA) and the number of microorganisms were decreased, and a negative influence on the fermentative profile was observed, although with an increase of *Bifidobacterium*. The ratio of butyric/propionic acids was also affected, indicating that those SCFA could affect the gut microbiota composition. Cyclamate also exerts a positive effect, producing an inhibitory anaerobic fermentation of glucose in a rat model of intestinal gut microbiota [28].

Cyclamate and sucralose can alter the ratio between butyric and propionic acids [30]. SCFA have multiple effects on human health. Butyric acid has anti-obesogenic effects, reduces insulin resistance, and improves dyslipidemia [31]. Lower concentrations of propionic and butyric acids have been positively correlated with the four subtypes of IBS and can be harmful to people with that disease [32]. Overall, it seems that cyclamate has some effects on gut microbiota composition, but more studies on its possible effect on human health are needed.

#### 2.1.4. Sucralose

Sucralose has a very low level of absorption (less than 15%) and it is practically not metabolized. Therefore, after intake, more than 85% of sucralose reaches the colon unchanged [10]. The small proportion of sucralose that is absorbed is eliminated in the urine mainly unchanged, though two glucuronides of sucralose were also detected in a small proportion (approximately 2%) [33].

Although more than 85% of the ingested sucralose contacts the colonic microbiota, between 94% and 99% of this LNCS is recovered in the feces without any structural change, thus indicating little or no metabolism by the gut microbiota [10]. Thus, sucralose does not appear to be a substrate for the colonic microbiota [16]. Nevertheless, considering the practically null microbial metabolism of sucralose, we must be cautious when interpreting the results of studies that indicate an alteration of the intestinal microbiota after sucralose consumption [17]. In those cases, it will be worth investigating whether pure sucralose or a commercial formulation was used in the research, since these formulations usually contain around 1% of sucralose and 99% of the carriers maltodextrins [16].

On the other hand, it has been shown that sucralose promotes inflammation in a mouse model of human Crohn’s disease-like ileitis as well as dysbiosis of the gut microbiota [34]. Furthermore, sucralose causes a decrease in the number of Firmicutes species [35]. This result is the opposite to that reporter by Olivier-Van Stichelen et al., who found that Firmicutes doubled, including the Clostridiales families Lachnospiraceae and Ruminococcaceae (e.g., Oscillospira), in mice’s pups [27]. Wang et al. observed an increase of Firmicutes and a tendency to decrease for Bacteroidetes [28]. These authors did not observe changes in Actinobacteria and Proteobacteria phyla in mice fed with a chow diet, but they reported a synergistic effect when sucralose was provided in the context of a high-fat diet. On the other hand, a chow diet might cause a significant increase in *Bifidobacterium* [28]. A study carried out in humans examined the short-term effect of sucralose consumption on glucose homeostasis and gut microbiome in healthy male volunteers. The authors concluded that no changes occurred in the gut microbiome due to sucralose intake [36]. In contrast, another study shows an increase in the abundance of pro-inflammatory bacteria like *Turicibacter*, which was associated with hepatic inflammation, after sucralose administration [37].

Splenda administration in mice was associated with a high presence of Bacteroidetes, an enhanced overgrowth of *E. coli*, and the expansion of Proteobacteria [38]. The effect of sucralose was analyzed in fecal samples from 13 healthy volunteers. The authors found increased abundances of *Escherichia*, *Shigella,* and *Bilophila.* With regard to SCFA, increased production of valeric acid was observed [19].

A recent publication evaluated the short-term effect of sucralose consumption on glycemic control and its interaction with the intestinal microbiota (comparison before/after the intervention by 16S rRNA sequencing) in healthy subjects. This study concluded that consumption of high doses of sucralose (75% of the ADI) for 7 days did not alter glycemic control, insulin resistance, or intestinal microbiome at the phylum level [36].

Although previous human studies showed similar results concerning glycemic control (glycosylated hemoglobin, fasting blood glucose, C-peptide), both in diabetic [39] and in non-diabetic populations [40], this is the first time that a randomized, controlled, double-blind study concomitantly evaluated the composition of the intestinal microbiome in healthy subjects, thus providing a better level of evidence in comparison to other earlier published trials.

#### 2.1.5. Saccharin

After intake, more than 85% of saccharin is absorbed as an intact molecule, since it does not undergo gastrointestinal metabolism. Once absorbed, it binds to plasma proteins and is distributed throughout the body. Finally, it is eliminated by urine through active tubular transport [10,41,42]. The small percentage of non-absorbed saccharin is excreted into the feces, indicating that high concentrations of this LNCS could lead to changes in the composition of the intestinal microbial population [16]. It is important to highlight that one of the main studies that reported an alteration of the intestinal microbiota with the consumption of saccharin [17] was carried out by administering the full ADI of saccharin, which does not correspond to what happens with habitual human consumption.

In an in vitro model study, saccharin produced an increase in *Bifidobacterium*. Not only saccharin but also sucralose caused a decrease in the number of Firmicutes species, directly correlated with the SCFA level [30].

Some herbicides, which are considered nowadays safe, can change the gut microbiota of animals in the early stages of embryonic development. Indeed, exposure to glyphosate and glyphosate in combination with saccharin contributes to the broader reproduction of pathogenic bacteria such as *Klebsiella*, *Citrobacter*, *Enterobacter,* and *Pseudomonas* [43]. On the other hand, studies show that saccharin administration can also disrupt monolayer integrity and alter paracellular permeability in a Caco-2 cell monolayer model [44].

Overall, saccharin administration also promotes Bacteroidetes, Turicibacter, and Clostridiales and reduces Firmicutes abundances. The Turicibacter bacteria increases have been related to a pro-inflammatory effect of saccharin [37].

The effect of a mixture of fiber–prebiotics and saccharin–eugenol has been evaluated in dogs. Four diets were prepared: control diet, containing 5% of cellulose; diet containing a 5% fiber and prebiotic blend; diet containing 0.02% of saccharin (sweetener SUCRAM) and eugenol and 5% of a fiber and prebiotic blend plus 0.02% of saccharin and eugenol. The use of saccharine did not affect species richness measured by alpha-diversity or alter the proportions of bacterial phyla. No changes were observed in fecal microbial communities [45]. More studies are needed to confirm these saccharin effects using different concentrations and animal models.

#### 2.1.6. Steviol Glycosides

Steviol glycosides can be extracted from the leaves of *Stevia rebaudiana*. They all have a central steviol structure, conjugated with different sugar residues, such as stevioside and rebaudioside A, which all are steviol glycosides. Steviol glycosides are hydrolyzed neither by enzymes nor by the acid present in the upper gastrointestinal tract [46]. Therefore, they pass through the upper portion of the gastrointestinal tract without being absorbed and enter the colon as intact molecules [47]. In the colon, bacteria of the Bacteroidacea family eliminate the sugar residues that are conjugated to steviol [47,48]. While these sugar residues may represent a source of energy for the microbiota [49], it is worth noting that the energy contribution is negligible, given the low total daily intake of steviol glycosides [50]. The resulting steviol is not a substrate for the intestinal microbiota, since it is resistant to bacterial degradation [48]. Hence, steviol is completely absorbed and reaches the liver where it is conjugated with glucuronic acid. Steviol glucuronide is mainly excreted in the urine in humans [51,52].

While steviol glycosides interact with the colonic microbiota, there are no reports indicating that these compounds could affect bacteria negatively [30]. A recent study showed that steviol incubation in the GIS1–phase 2 system, an in vitro system that simulates the human intestinal microbial ecosystem, reduced the ammonium level and *Bifidobacterium* and exerted a negative influence on the fermentative profile, resulting in higher pH and SCFA ratio [30].

*S. rebaudiana* is another natural steviol glycoside 250 times sweeter than sucrose [53]. In Europe, only the purified steviol glycosides are approved for use in food, and the ADI of 4 mg kg^−1^ of body weight per day is safe (EU Regulation (EU) 1129/2011) [1,7,54]. Another study recently reported that a low dose of stevia rebaudioside A alters gut microbiota composition and reduces nucleus accumbens tyrosine hydroxylase and dopamine transporter mRNA levels in rebaudioside A-supplemented rats. Nonetheless, the oligofructose-enriched inulin prebiotic, in the presence or absence of rebaudioside A, reduced fat mass, food intake, gut permeability, and cecal SCFA concentration. However, only stevia rebaudioside A increased SCFAs acetate and valerate, which are positively correlated with fat mass and total weight. Hence, stevia rebaudioside A seems to decrease the “healthy” status of the gut microbiota [55].

Chronic stevia consumption has effects on gut microbiota and immunity in the small intestine of young mice. In 21-day-old mice treated with sucrose, Splenda, and stevia, mice preferred the consumption of Splenda and stevia. Besides, those mice showed an increase in CD3^+^ lymphocytes in Peyer’s patches, but only stevia induced an increase in the lamina propria. Both Splenda and stevia elevated leptin, C-peptide, IL-6, and IL-17 and decreased resistin. Stevia modified the predominantly genera *Bacillus* such as *Bacillus aerius*, *Bacillus circulans*, *Bacillus licheniformis*, and *Bacillus safensis*, although the authors observed effects on *Streptococcus saliviloxodontae*, *Oceanobacillus sojae*, and *Staphylococcus lugdunensis*. Even though the results of this study are significant, they have some limitations. The modifications observed in the immune system of the mucous membranes and in the microbiota of the small intestine in young mice after weaning depend on age and diet. This study used culture media and not metagenomic approaches, and some results might be related to some carriers present in the evaluated products, such as maltodextrins [56].

Recently, by testing stevia glycosides and erythritol, which are often combined in food preparation to minimize changes in the organoleptic profile, in an in vivo *Cebus apella* model, changes in bacteria growth and gut microbial structure and diversity have been observed [57]. Overall, stevia seems to modify the gut microbiota; however, further studies are needed to clarify its specific effects.

Although different changes in the intestinal microbiota have been described in relation to the influence of sweeteners on the immune system, the wide use of aspartame, ace-K, cyclamate, sucralose, saccharin and steviol glycosides makes it necessary to carry out other analyses to complete the picture of the influence that these sweeteners have on the intestinal microbiota.

### 2.2. Effects of Nutritive Low-Calorie Sweeteners on the Gut Microbiota

#### 2.2.1. Polyols

Polyols are a group of compounds used in an increasingly wide variety of commercial foods as additives. They are quite stable at high temperatures and various pH and do not interfere in Maillard reactions, conferring organoleptic characteristics to the foods. Polyols are naturally present in fruits, vegetables, and mushrooms and are used to produce food without added sugar, reducing the sugar content in recipes. In addition, polyols are non-cariogenic, do not induce salivation, and do not interfere with insulin and glucose levels in the blood. Nevertheless, the excessive consumption of polyols causes gastrointestinal symptoms and laxative effects, which can be even worse in patients with IBS. As we described previously, the FDA, the Codex Alimentarius, and the EFSA have approved eight different polyols, i.e., erythritol, hydrogenated starch hydrolysates, isomalt, lactitol, maltitol, mannitol, sorbitol, and xylitol, for use as bulk sweeteners in human foods [1,58]. Indeed, in September 2019, EFSA launched an open consultation on the “Protocol for the assessment of hazard identification and characterization of the sweeteners”, which will be used for the evaluation of the safety of sweeteners under the re-evaluation program of food additives. The evaluation should be completed by the end of 2020 [59].

#### 2.2.2. Erythritol

Erythritol (E-968) is a four-carbon sugar alcohol that has a fast absorption through the small intestine with a very low metabolization and it is over 90% excreted unchanged in the urine [58]. Furthermore, an unabsorbed part (~10%) is fermented in the large intestine by the colonic microbiota, which consequently rarely leads to gas production [60]. Hence, the limited amount of erythritol that reaches the colon could be the explanation of the lack of evidence of effects of erythritol on the gut microbiota in humans, based on clinical trials as we previously reported [1]. Nevertheless, a recent in vitro study demonstrated that low doses of erythritol (25 μg mL^−1^, 50 μg mL^−1^, and 100 μg mL^−1^) did not exert any effect on the growth of *Escherichia*, *Enterococcus*, *Lactobacillus*, *Ruminococcus* and Bacteroides in the human gut microbiota. Moreover, erythritol doses did not disrupt alpha and beta diversities or the composition of the human gut microbial community [57]. In contrast, butyric and pentanoic acids were increased significantly after erythritol consumption, indicating that this polyol may be able to affect the function of the human gut microbiota. Indeed, the authors reported that this change in SCFAs production was due to the 10% of erythritol that reaches the human colon [57].

#### 2.2.3. Isomalt

Hydrogenated isomalt, isomaltitol (E-953), is not absorbed by the small intestine and is easily fermented in the colon by the microbiota [61]. This fermented fraction of ingested isomalt is approximately 90% [62]. Therefore, it is expected that isomalt is capable of altering the bacterial population. Isomalt has been proposed as a prebiotic carbohydrate that might contribute to a healthy luminal colonic mucosal environment, with bifidogenic properties and high butyrate production [63]. Accordingly, besides evidence reported in Ruiz-Ojeda et al., 2019 [1], a recent study based on the administration of buckwheat honey to human gut microbes cultures reported that the principal constituents of buckwheat honey are oligosaccharides with a low degree of polymerization, including isomalt and isomaltotriose, which may serve as food to promote the growth of indigenous intestinal probiotics such as *Bifidobacterium* [64]. In addition, an increase in the abundance of *Escherichia*/*Shigella* and *Streptococcus* was also reported, while the alpha diversity, as well as the abundance of *Prevotella*, *Faecalibacterium*, and *Lachnospiraceae incertae sedis*, were decreased, thus fostering a reduction of pathogenic bacteria in the gut tract [64]. However, this might be also explained by the polyphenols composition of the buckwheat honey studied, since polyphenols also markedly affect the gut microbiota [64]. Indeed, the authors concluded that phenolic compounds and oligosaccharides in buckwheat honey appear to synergistically impact human intestinal microbes to enhance the growth of probiotics. More efforts, especially in vivo, are required to elucidate the possible specific impact of isomalt on the gut microbiota.

#### 2.2.4. Lactitol

Lactitol (E-966) is a disaccharide normally not absorbed in the small intestine [65] that therefore reaches the lower gut where it is fermented, producing both gases and SCFA [66]. Lactitol mitigates pathogenic translocation in the small intestine by the reduction of permeability and stimulates the growth of bifidobacteria and lactobacilli [67]. Thus, similarly to isomalt, lactitol could act as a prebiotic, enhancing the composition of the intestinal microbiota, even when consumed at low doses as a sweetener, normally 10 grams [68]. Nevertheless, it is important to highlight that lactitol, due to its limited sweetening power, is usually used in combination with other intense sweeteners [69] or a set of prebiotics [70], and this could disturb the results concerning its effect on the intestinal microbiota. Furthermore, it has also been studied as a synbiotic product along with *Lactobacillus acidophilus* NCFM and jointly promoted beneficial changes since it led to a decrease in the abundance of the *Blautia coccoides*–*Eubacterium rectale* bacterial group and *Clostridium* cluster XIVab counts in the elderly population [71]. Since 2018, two studies were identified regarding lactitol and the gut microbiota. One trial was based on the administration of probiotics, synbiotics, probiotics together with lactitol, or only lactitol to mice with acute colitis. The authors found that the lactitol group showed higher levels of *Akkermansia* compared with the control, probiotic (*Bifidobacterium* and *Lactobacillus*), and synbiotic (probiotics and inulin) groups. It is worth highlighting this work, since *Akkermansia* seems to ameliorate the inflammatory response and insulin resistance in obese and diabetic patients [72], protecting the intestinal epithelial cells and enhancing the mucosal barrier function [73]. As the genome of *Akkermansia* was proved to be able to encode a wide variety of secretory proteins such as glycohydrolyzases [74], the authors speculated that *Akkermansia* might be able to decompose lactitol and promote its own proliferation [75]. Furthermore, the supplementation of probiotics and prebiotics, including lactitol, induced an increment of the proportion of helpful bacteria and regulated the balance of the intestinal microbiota [75]. For instance, the abundance of *Bifidobacterium* was increased in all the experimental groups in comparison with the control. However, the observed effect might not be exerted by lactitol itself [75]. Another study was performed in Korean adults to evaluate the efficacy of supplementation with the prebiotic UG1601 (based on inulin (61.5%), lactitol (34.6%), and an aloe vera gel (3.9%)) for 4 weeks to alleviate the symptoms of constipation associated with the gut microbiota [70]. Here, the clinical trial showed that the prebiotic UG1601 in patients with mild constipation resulted in decreased serum concentrations of the bacterial endotoxin lipopolysaccharide and its receptor CD 14. Additionally, it increased the abundance of *Roseburia hominis*, a major butyrate producer, which could be related to the observed reduction of the levels of these endotoxemia markers [70]. In summary, lactitol along with other compounds, may induce changes in the gut microbiota, but further studies are needed to demonstrate whether lactitol itself triggers an effect on the gut microbiota.

#### 2.2.5. Maltitol

Maltitol (E-965) is obtained through the hydrolysis, reduction, and hydrogenation of starch. This polyol has a very slow absorption rate, being fermented in the colon. Thus, as we previously mentioned, it is expected that maltitol is susceptible fermentation by the gut microbiota [1]. To date, only one clinical trial has been reported which studied the effect of maltitol present in experimental chocolate on the gut microbiota. The authors concluded that both maltitol and polydextrose, as well as maltitol alone, increased the amount of fecal bifidobacteria, lactobacilli, and SCFA compared with the control chocolate [26]. Besides evidence reported by Ruiz-Ojeda et al., 2019 [1], there are no additional studies. Although maltitol could be a good alternative with high sweetening capacity (~90%), safe, and non-cariogenic, data to determine the specific effects of maltitol on the gut microbiota are not still sufficient.

#### 2.2.6. Sorbitol

Sorbitol or D-glucitol (E-420) is partially absorbed in the upper gastrointestinal tract, where it undergoes digestion, while the non-absorbed portion is extensively fermented to SCFA and gases by the colonic microbiota [62]. Consumers can suffer slight gastrointestinal symptoms, such as flatulence or bloating, or more severe symptoms when it is ingested at high doses as 20g d^−1^ [76]. Overall, studies on this isomeric polyol and its effect on the gastrointestinal tract are mostly focused on the symptomatology induced by sorbitol than on its possible capacity to alter the gut microbiota. Since the 1930s, it is known that sorbitol can be fermented by bacteria like *E. coli*, *Lactobacillus* spp., and *Streptococcus* spp. [77] which are present in our intestinal microbiota. However, so far, there has been no thorough study and there is not enough evidence to define the specific effects of sorbitol on the gut microbiota.

#### 2.2.7. Mannitol

Mannitol (E-421) is an isomer of sorbitol, and both are listed as hydrogenated monosaccharides. Approximately, 75% of ingested mannitol reaches the large intestine [78]. The intestinal bacteria metabolize D-mannitol to butyrate and propionate in animal models. Indeed, D-mannitol has been suggested as a prebiotic, due to its stimulation of colonic butyrate and propionate production [79]. Although no data are available so far about the effects of mannitol on the gut microbiota, its role as a substrate reflects an interaction between this polyol and the intestinal microbiota that should be studied more deeply.

#### 2.2.8. Xylitol

Xylitol (E-967) is a five-carbon polyol obtained from the hydrogenation of D-xylose, called wood sugar or birch sugar. Xylitol can be directly metabolized mainly in the liver, remaining unchanged in the gastrointestinal tract [80]. Furthermore, only a certain proportion of the ingested xylitol is absorbed slowly from the intestinal lumen and fermented by the intestinal microbiota. Besides minor amounts of gases such as H_2_, CH_4_, and CO_2_, the end products of the bacterial metabolism of xylitol are mainly SCFA, (i.e., acetate, propionate, and butyrate). Xylitol might cause osmotic diarrhea when the amounts consumed are too high [81]. Hence, it is expected that this polyol is capable of altering the intestinal microbiota. Interesting results were reported, as previously mentioned, in our recent review [1], but further studies were not reported since then.

**Figure 1:**
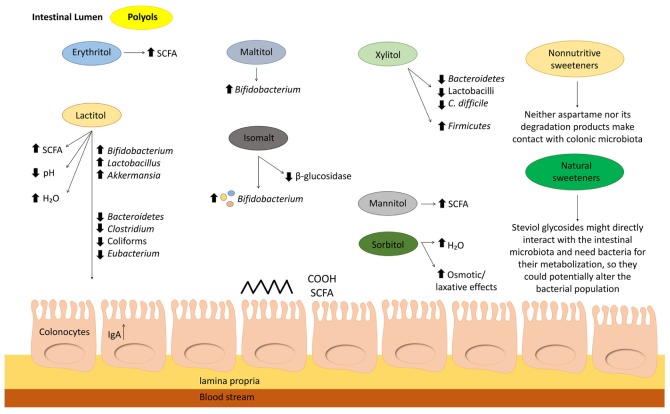
Schematic representation of sweeteners’ effects on the gut microbiota. Abbreviations. IgA, Immunoglobulin A; N/A, not available information; SCFA, short-chain fatty acids.

In summary, according to the new findings reported from February 2018, erythritol, lactitol, and maltitol have shown to exert beneficial effects on the gut microbiota by themselves. Nevertheless, because of the promising effect of lactitol to enhance *Akkermansia* proliferation in mice with acute colitis, we encourage corroborating this finding by further studies in humans. Overall, the latest evidence is not still enough to establish firm conclusions in relation to how polyols influence the gut microbiota. In addition, it is necessary to highlight that some polyols could induce laxative effects, and it would be more reliable to evaluate their effects separately. Figure 1 summarizes the effects of different sweeteners on intestinal microbiota.

## 3. Conclusions and Future Perspectives

The effects of sweeteners on gut microbiota composition are still in discussion. Even though there are some gaps in the evidence related to the health effects of NNS in both healthy and non-healthy populations, authorities such as FDA, EFSA, and Codex Alimentarius consider them safe and well-tolerated, as long as the appropriate ADI is not exceeded. Regarding NNS, neither aspartame nor its degradation products make contact with the colonic microbiota. In contrast, though Ace-K is absorbed and eliminated by urine and almost does not contact the colonic microbiota, surprisingly, it increases Firmicutes and depletes *A. muciniphila.* However, further research is required in order to firmly establish an effect in humans. We previously reported that saccharin and sucralose seem to change the composition of the gut microbiota. However, it is necessary to take account that only 15% of the consumed saccharin contacts the colonic microbiota, so only high doses could alter the intestinal microbiota composition. On the contrary, more than 85% of the consumed sucralose reaches the colon; therefore, sucralose could potentially either alter or change the gut microbiota composition, but it is not practically metabolized by intestinal bacteria. On the other hand, steviol glycosides directly interact with the intestinal microbiota and need bacteria for their metabolization, so they could potentially alter the bacterial population.

In summary, in the absence of biological plausibility, results indicating a possible alteration of the intestinal bacteria population after the consumption of LNCS should be explained by alternative mechanisms, such as alterations in the dietary pattern, administration of exaggerated LNCS doses, and co-administration of carriers.
